# Editorial Department

**Published:** 1856-02

**Authors:** 


A writer in the St. Louis Journal, over the signature of "An Old Fogy," whose article we are half-inclined to publish for its pith and raciness, though we dissent roundly from its conclusions, asks "what are the direct facts as to the utility of the microscope in the diagnosis, say of tumors for example? Why, the facts show that the microscope is of just about no service at all." Now "An Old Fogy" might quite as well assert that no eyes at all were as good as the sharpest optics of Young America. In "tumors for example," we are ready to admit that there has been a good deal of hasty conclusion; but a little instance occurring the other day within our knowledge, is worth pages of argument in this matter. A patient had a discharge from the urethra. A physician of large experience and excellent judgment, called it spermatorrhœa, and proposed the porte-caustic. Another physician, of small experience and a microscope, tested the discharge. It had no spermatozoa, but it did have an abundance of pavement epithelium in it—Q. E. D. The discharge was not spermatorrhœa, and its source was confined to the first inch and a half of the urethra, or he would have found spheroidal cells. Here was a diagnosis of considerable importance, which only the microscope could have made out. Again, how would "An Old Fogy" have known, without the microscope, that there are such things as spermatozoa, spheroidal or flattened cells? We pause for a reply!

